# The Testing of Drains

**Published:** 1903-01-03

**Authors:** 


### THE TESTING OF DRAINS.

The subject before the Sessional Meeting of the Sanitary Institute on December 10th was "Drain-Testing." The chair was taken by Professor W. H. Corfield, M.A., M.D., F.R.C.P., Vice-President, and about 150 persons—half a dozen of whom were ladies—were present. The discussion was opened by Dr. Louis Parkes, Medical Officer of Health for Chelsea, who outlined the statutory powers of inspectors under the Public Health Acts. It would appear that whilst there is no express authorisation for the testing of drains, there is absolutely nothing in the shape of any veto. Inferentially, tests would seem to be sanctioned, as otherwise how could the local authority perform its statutory duty of ascertaining if the drains of its district are in good order or not? The main question for discussion, he thought, was whether the interests of the public health demanded that all drains should be water-tight. If this question were answered in the affirmative, the next point to settle was whether the existing statutes conferred the necessary powers to enable sanitary authorities to compel owners to relay all non-water-tight drains, even if found to be unproductive of nuisance at the time of examination. If the general consensus of opinion was that such drains should be relaid, and that the existing statutes did not give the necessary powers, then fresh legislation would be required to meet the demand.

Mr. J. Osborne Smith, F.R.I.B.A., followed. In dealing with new buildings, he said, the treatment of drainage was comparatively simple and direct; the work should be absolutely water-tight, and capable of resisting any hydraulic pressure likely to arise in practice. The risk of harm arising from very small leaks in well-constructed drains was however comparatively slight, and the experience of the Medical Officers of Health would enable them to indicate to what extent leakage from a drain, say three to six feet below the surface of the ground-level and outside a building, might be injurious or dangerous to health. No hard and fast rules could be laid down as to testing the efficiency of old drainage. Haphazard guessing from meagre data on the one hand, and reckless application of rigorous, destructive tests on the other, were equally to be deplored. "We have gone far," he remarked, "in the education of the general public in appreciation of the importance of sanitary science. We must beware lest, by the extravagance of specialism, we provoke a reaction."

The third "opening" speech was by Mr. W. C. Tyndale, M.Inst.C.E. Since 1876 it had been generally recognised that foul-water drains should be water-tight, and the law was clear as far as new work was concerned. The difficulties, however, connected with the testing of an existing drainage system were numerous, since so many interests were concerned, *e.g.*, those of the landlord, occupier, public, doctors, and engineers, and the great difficulty lay in arriving at what was right and proper from the health point of view, and, at the same time not unfair in other directions. It would save much trouble and hardship if local authorities would observe a little latitude in particular cases, and accept the opinion of an expert of repute instead of always standing out for absolute water-tightness. Although he resented the tyranny of bye-laws, in his opinion the only way to insure healthy conditions, and to meet the difficulties they were met to consider, was for local authorities to insist on all house drains being laid with iron pipes.

Eight or nine gentlemen took part in the discussion, most of them, apparently, being sanitary inspectors. The opinion that an equal standard of water-tightness should be demanded of new drains and old was loudly applauded, and the Chief Inspector for Battersea (Mr. Isaac Young) hoped the meeting would not separate without some definite pronouncement on this important point. When it was stated by some health officers that it was unreasonable to expect old drains to stand the water-test, he thought (and the meeting evidently agreed) that it was time to have the old drains "ripped up." Another inspector said he had come to hear whether a drain should be water-tight, old or new, and he very much regretted that the Institute had not told him what a good drain should be. Was a defective drain a nuisance or not? He asked the Institute to give a definite answer. (Great applause.)

When does a drain end and a sewer begin? was another question. Why was the eye of the sewer not tested in the same way as the drain? What *class* of old drain should be permitted to pass, and what not? "What is a good drain?" a voice asked from the gallery, when the discussion had proceeded on these lines for some time. "At what point is a sanitary inspector to condemn a drain? Again, if he passes a drain, and it afterwards becomes stopped and causes somebody's death, whose fault is it?" One speaker said it was all a question of cost; another that the inspector's duty was to administer the Public Health Acts, and cost ought not to affect the matter. The opinion that the smoke test was a waste of time drew forth applause. Was it not absolutely foolish to apply it to drains with six inches of concrete round them?

Several speakers were in favour of iron pipes, and one said that the sooner the architects gave their opinion on this point the better it would be for the inspectors. As to when drains should be examined, Dr. MacLeod said that besides being tested when the house was built, the process should be repeated before occupation, and again when the made soil had subsided. Railway cuttings frequently dislocated pipes and loosened joints, and there should be testing in such cases.

The Chairman, in reply to some of the questions, said some sanitary authorities did inspect the part of the drain between the interception and the sewer ("Many don't," said several voices). He advised the testing of new drains when laid, after covering in with concrete, and again some time afterwards. The smoke test was a most useful one for ascertaining whether a drain was unsound, but no use whatever for finding out whether it was sound. If the drain did not hold smoke, it was obviously unnecessary to apply the water test. Did the water test damage drains? He believed it was a pure bogey (prolonged applause). It would damage drains laid with clay joints; they could be made water-tight, but how long would they remain so? If a defect existed, the water test might exaggerate it, and the sooner the better. It was contrary to sound sanitary practice to take a material known to be destructible, and try to coat it with something, and then hope it would last. Stoneware remained cleaner inside, and he would prefer it on account of its unalterableness, were it not for the unfortunate fact that it was not always sound after a year. Therefore it would probably be agreed that drains under a house were better made of heavy iron pipes. In his opinion, all new soil pipes should be tested with water, and in some instances old ones; but the chemical or smoke test was usually sufficient in these cases. If the rain pipes were at all connected with the soil pipes, they also must be tested.

On the motion of Mr. Isaac Young, the following resolution was unanimously passed: "That this meeting of the Sanitary Institute is of opinion that all systems of drainage for conveying sewage should be capable of resisting a pressure of at least two-feet head of water."

